# Leveraging Artificial Intelligence to Bridge the Gap Between Evidence and Practice in Cardiology

**DOI:** 10.1016/j.jacadv.2025.101851

**Published:** 2025-06-11

**Authors:** Rohan G. Reddy, David A. Danford, Shelby Kutty

**Affiliations:** a The Johns Hopkins University, Baltimore, Maryland, USA; b BayCare Health System, Clearwater, Florida, USA

**Keywords:** artificial intelligence, evidence-based practices, implementation science

Recent clinical science advances in cardiology have resulted in new evidence-based practices (EBPs) both in diagnostics, like artificial intelligence-guided cardiovascular imaging techniques, and in therapeutics, like treatments for heart failure and arrhythmias. Nevertheless, the translation of EBPs into routine clinical care remains sluggish, resulting in a research-to-practice implementation gap. For example, for patients with heart failure with reduced ejection fraction (HFrEF), guideline-directed medical therapy (GDMT) has been shown to reduce HFrEF-related morbidity and mortality, and yet its implementation lags in actual practice.^1^ Due to the slow pace of integrating EBPs into clinical practice, there has been a growing interest in implementation science (ISci), which is dedicated to identifying methods by which closure of the implementation gap may be optimized and accelerated. To date, there have been few studies of ISci strategies in cardiovascular medicine to promote the uptake of EBPs, possibly because conventional ISci methods are resource-intensive and time-intensive, and therefore somewhat daunting.^2^ We believe artificial intelligence (AI) algorithms can expedite the translation of scientific findings to clinical practice, thus reducing the research-to-practice gap. AI can be integrated into 4 commonly utilized ISci steps: 1) identifying an EBP of interest; 2) identifying barriers and facilitators for its use; 3) designing an implementation strategy; and 4) testing the implementation strategy (Table 1).^3^ To illustrate the potential of AI for improving these areas of ISci, we will focus on the GDMT implementation gap in HFrEF.

**Table 1:** Comparison of Traditional Versus AI-Augmented ISci Steps

ISci Steps	Traditional Methods	AI-Assisted Methods
1. Identify the evidence-based practice of interest	• Manual EHR reviews • Surveys with stakeholders	• ML-assisted EHR reviews • NLP analysis of patient notes
2. Identify barriers and facilitators for EBP use	• Use of ISci frameworks (ie, CFIR, RE-AIM, etc) • Focus groups • Semi-structured interviews	• Focus groups/interviews with generative AI • Predictive AI-models
3. Design an implementation strategy	• Educational programs • Audit and feedback • Incentives	• Real-time AI-driven personalized feedback • AI models designed to provide culturally competent and tailored educational materials
4. Test the implementation strategy	• Implementation trials	• Implementation trials supported by AI-driven real-time data dashboards • Predictive AI-models

AI = artificial intelligence; CFIR = Consolidated Framework for Implementation Research; EBP = evidence-based practice; EHR = electronic health record; ISci = implementation science; ML = machine learning; NLP = natural language processing; RE-AIM = Reach, Effectiveness, Adoption, Implementation, and Maintenance.

## Identifying the evidence-based practice of interest

Identifying an evidence-practice gap is the first step of ISci frameworks and traditionally begins with quantitative and qualitative assessments. Electronic health record (EHR) data are reviewed quantitatively and analyzed for clinical outcomes, guideline adherence, and prescription rates, and this is often followed by manual audits of individual patient charts and notes to pick up nuances that may not have been readily captured in the initial analysis. Qualitative surveys and assessments may be performed with patient caregivers, including physicians, patients, and leadership, to better understand where these evidence-practice gaps lie.^4^ For example, implementation scientists may review EHR data to analyze prescription patterns for patients with HFrEF and compare the data with the American Heart Association guidelines to see if the prescription rates indicate GDMT is being delivered. Their surveys and interviews with key stakeholders like cardiologists, internists, and pharmacists may provide insight into their perspectives and practices when treating patients with HFrEF. While these strategies can be very useful in identifying an implementation gap, they are time- and resource-intensive. As a result, these methods are generally not ongoing or continuously updated, so they may not accurately capture the changing nature of clinical practice as new interventions or contexts emerge.^5^

AI can improve the efficiency of EBP gap identification by automating the analysis of EHR data. For example, machine learning (ML) algorithms can be applied to EHR data to analyze physician practices like prescription rates to see if they deviate from guideline recommendations. Specifically, ML models could theoretically evaluate prescription patterns of angiotensin receptor-neprilysin inhibitors, beta-blockers, mineralocorticoid receptor antagonists, and sodium-glucose cotransporter-2 inhibitors among eligible patients with HFrEF to more efficiently identify and characterize implementation gaps. Additionally, natural language processing (NLP) can analyze unstructured data in clinician notes to reduce the need for time-consuming and labor-intensive manual audits.^2^ These methods can be applied to many cardiac conditions to identify EBP gaps more efficiently than traditional ISci strategies.

## Identifying barriers and facilitators for use

Once an EBP gap is identified, the search for barriers and facilitators begins by applying well-known ISci frameworks like the Consolidated Framework for Implementation Research. By drawing upon mixed or qualitative methods like focus groups and semistructured interviews with physicians and other medical staff, Consolidated Framework for Implementation Research gauges their perspectives on why a specific implementation gap may exist and challenges they may face.^6^ However, these methods are often subjective, time-intensive, and low-powered.^4^ Generative AI can automate and enhance this step through natural language interactions with caregivers individually or in focus groups, which can then be performed concurrently, saving time and resources.^2^ AI can also analyze EHR data to link clinician practices to the underlying factors that influence them. For example, in patients with HFrEF, ML models can identify variables that predict suboptimal prescription of GDMTs, which can then guide future implementation strategies.^7^ Beyond HFrEF, such models could also be adopted to identify barriers in other areas of cardiology practice with implementation gaps, such as the use of AI-guided cardiovascular imaging models or ML-driven risk stratification models for patients with severe aortic regurgitation. Moreover, NLP can further assess clinical notes for factors contributing to clinician inertia that obstruct the implementation of EBPs.

## Designing an implementation strategy

After identifying barriers and facilitators surrounding EBP implementation, the next step is to design an effective implementation strategy on both the individual caregiver level and the health care system level. Strategies often include educational programs, audit and feedback, and providing incentives. While some of these strategies can be effective, they usually take long periods of time to implement and even longer to assess. This is a major limitation because the context of clinical practice is continually evolving, but AI can help propel implementation strategies by offering a versatile, personalized, and adaptive approach. This great advantage of AI is based on algorithms that constantly operate to incorporate new data and evolve quickly to changing circumstances.^2^

In the context of GDMT for HFrEF, the IMPROVE HF study illustrated that performance improvement measures like clinical decision support, EHR reminders, and patient education could increase the prescription of GDMT at 24-month follow-up.^8^ Moreover, the established Get With The Guidelines-Heart Failure hospital-based quality improvement program has also been shown to improve guideline compliance rates for participating hospitals through feedback reports and benchmarking.^9^ Moreover, traditional audits and feedback in the form of physician reports have been effective in various studies to improve prescription rates.^10^ However, they are still retrospective and manual processes that can lead to delays, directly affecting patient outcomes.^1^ Using AI to monitor patient charts in real time could provide faster feedback. Using NLP to provide more personalized and targeted feedback by analyzing clinician notes is an appealing alternative to less efficient manual audit and feedback programs.

The power of personalization can be a great advantage of AI models in ISci. By learning physicians' prescription patterns and providing EHR reminders in impactful moments during patient care, AI potentially maximizes the likelihood of successful prescription of GDMTs where appropriate.^1^ Beyond physician-level interventions, AI models can also provide efficient patient education, which has been shown to improve GDMT intensification.^8^ AI models could provide personalized educational materials based on patients’ unique histories and backgrounds while also using translation abilities to offer culturally competent information to patients.^2^ The scope of these AI applications extends well beyond HFrEF to other areas of general cardiology where EHR reminders, audit and feedback, and personalized educational materials are anticipated to be useful.

## Testing the implementation strategy

The final step after designing an implementation strategy is to test the strategy’s sustainability and its effectiveness to facilitate integration of the EBP into real-world practice. To do so, traditional ISci conducts trials to evaluate the outcomes that follow implementation. It may also run effectiveness-implementation hybrid trials to evaluate the implementation strategy in conjunction with the efficacy of the actual EBP.^3^ While implementation trials are crucial, they often take a long time to complete and are costly, and therefore may not be very adaptive or flexible enough to make changes as contexts or circumstances shift. AI assistance can be advantageous because it analyzes and monitors data in real time, thus providing research staff with dynamic dashboards containing up-to-date data so that necessary changes to study design can be made rapidly. In addition, AI’s predictive capabilities can estimate the sustainability of strategies and predict factors that may influence it so that planning and resource allocation are better informed. For example, if workflow issues arise late after implementation, such as drifting or delayed prescription patterns, these could be quickly captured with AI assistance, and changes like adjusting the frequency or timing of EHR reminders could be made promptly. Implementation trials will remain a crucial and necessary step in ISci, but AI could help improve their responsiveness and efficiency.

### Challenges to utilizing AI

Despite AI’s promise in ISci, it must be adopted cautiously with transparent acknowledgment of its possible negative consequences. Inaccurate or biased results can be generated by AI models if they are based on biased training data that fail to equitably represent the population.^2^ Thus, AI models must be trained and validated with data from diverse patient populations. Moreover, AI models can misinterpret data and thus provide misguided recommendations, which can be difficult to pinpoint if models are “black boxes,” such that the rationale behind the model’s decision-making process is unclear or unexplainable.^2^ For AI models to gain user trust and become widely implemented, the transparency of the methods that lead to an output is key.

## Conclusions

Much work must be done before AI can safely and effectively complement ISci. Still, we believe efforts to integrate AI into ISci are worthwhile because it will ultimately help bridge the gap between evidence and cardiology practice.

## Funding support and author disclosures

The authors have reported that they have no relationships relevant to the contents of this paper to disclose.
